# Lightweight Microcontroller with Parallelized ECC-Based Code Memory Protection Unit for Robust Instruction Execution in Smart Sensors

**DOI:** 10.3390/s21165508

**Published:** 2021-08-16

**Authors:** Myeongjin Kang, Daejin Park

**Affiliations:** 1 School of Electronic and Electrical Engineering, Kyungpook National University, Daegu 41566, Korea; audwls3158@knu.ac.kr; 2 School of Electronics Engineering and School of Electronic and Electrical Engineering, Kyungpook National University, Daegu 41566, Korea

**Keywords:** embedded system, parallelized execution, ECC, performance improvement

## Abstract

Embedded systems typically operate in harsh environments, such as where there is external shock, insufficient power, or an obsolete sensor after the replacement cycle. Despite these harsh environments, embedded systems require data integrity for accurate operation. Unintended data changes can cause a serious error in reduced instruction set computer (RISC)-based small embedded systems. For instance, if communication is performed on an edge, where there is insufficient power supply, the peak threshold is not reached, resulting in data transmission failure or incorrect data transmission. To ensure data integrity, we use an error-correcting code (ECC), which can detect and correct errors. The ECC parity bit and data are stored together using additional ECC memory, and the original data are extracted through the ECC decoding process. The process of extracting the original data is executed in the instruction fetch stage, where a bottleneck appears in the RISC-based structure. When the ECC decoding process is executed in the bottleneck, the instruction fetch stage increases the instruction fetch time and significantly reduces the overall performance. In this study, we attempt to minimize the effect of ECC on the transmission speed by executing the ECC decoding process in parallel to improve speed by degrading the bottleneck. To evaluate the performance of a parallelized ECC decoding block, we applied the proposed method to the tiny processing unit (TPU) with a RISC-based von Neumann structure and compared memory usage, speed, and reliability according to different transmission success rates in each model. The experiment was conducted using a benchmark that repeatedly executed several 3*3 matrix calculations, and reliability improvement was compared by corrupting the stored random date to confirm the reliability of the transmission success rate. As a result, in the proposed model, using the additional parity bits for parallel processing, memory usage increased by 10 bits per instruction, reducing the data rate from 80 to 61%. However, it showed an improvement in overall reliability and a 7% increase in speed.

## 1. Introduction

Currently, with the advancements in the computing power of embedded systems, many embedded systems can be operated in harsh external environments. However, environments in which signals are affected by an external shock or insufficient power supply can cause unwanted data damage in embedded systems [1]. A small 1-bit error in the operation of an embedded system causes a variety of errors, from simple calculation errors to critical system errors. To prevent this, embedded systems use error-correcting code (ECC) to protect data from unwanted data errors.

The data of the embedded system consists of 0s and 1s, but to store and transmit these data, voltages suitable for 0s and 1s are used. If external noise or impact is introduced while updating or transmitting the stored data, the data value is read incorrectly or becomes unreadable [2]. In this case, embedded systems can use ECC to protect the data and restore corrupted data [3]. There are several ECC algorithms to protect and restore damaged data [4,5]. There is simply a 1-parity method that counts and uses the number of 1 and a repetition method that acquires data with the highest number of repetitions by repeatedly transmitting the data several times. The 1-parity method can reduce the load on the tiny processing unit (TPU), but it cannot correct errors, though it can identify the error, wait for a correction, or proceed to the next step with corrupted data. Although the repetition method shows high accuracy, it cannot be used when data in the memory are corrupted, and because it has a very low data rate, the TPU has the disadvantage of processing (receiving and transmitting) a large amount of data. Among several ECC algorithms, the Hamming Code is commonly used in embedded systems [6,7] because single error correction double error detection (SECDED) is possible in one unit of data with a short additional parity bit [8]. The Hamming code algorithm generates parity bits through exclusive-OR (XOR) operation of data, stores the data, and verifies the integrity of the data through the decoding process before using the data [9,10,11]. In this process, when one error occurs, it can be corrected, and when two or more errors occur, it can be detected and retransmitted or users will be warned. The ECC process to be applied to the embedded system is similar to the SOTA design. It can be used by dynamically changing the operation itself in response to the changing situation, and it can be applied to the design and development of the ECC process according to the system’s situation [12].

**Figure 1:**
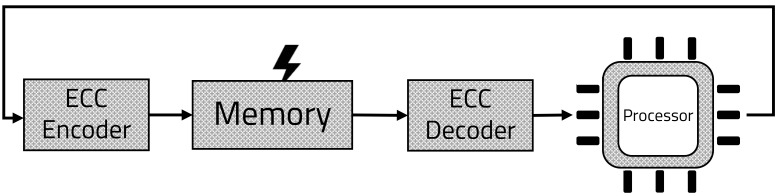
Ensure integrity of memory.

**Figure 2:**
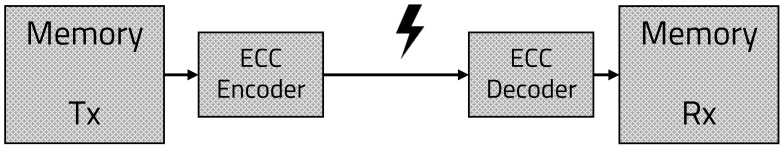
Ensure integrity of transmission process.

To ensure data integrity, the ECC processing block is placed in front of and behind the memory, as shown in Figure 1. To check the integrity of data in memory, the ECC encoding block will be located in front of the memory, and the decoding block will be located behind the memory. In this process, data corruption in the memory can be excluded by checking the parity of the data from the memory [13,14,15]. Conversely, the ECC decoding block is located before the memory. In this case, as shown in Figure 2, integrity of the data transmission process is guaranteed, because of the parity checking process after data transmission [16,17,18].

However, for embedded systems that lack computing power, the process of adding additional parity bits to data and ensuring integrity through computation can be a heavy burden. Adding encoding and decoding blocks can cause problems in the design size, speed, and power consumption [19]. In addition, the ECC decoding block can have a negative effect on affect processing speed. In the TPU’s von Neumann architecture, insufficient memory speed remains a bottleneck [20]. In this case, the ECC decoding block is located in the memory, worsening the bottleneck. One way to eliminate this bottleneck is to use a cache. Two levels of caches can be used on the microprocessor to speed up ECC processing. However, when a cache miss occurs, the ECC decoding process delays the fetch of the instructions, causing a full delay, which degrades performance.

When designing the TPU, the processing speed of the TPU is an important factor. In particular, when designing a small reduced instruction set computer (RISC)-based processor, the bottleneck significantly affects the output performance. From a hardware design viewpoint, if clock acceleration is possible, removing bottlenecks often improves the overall performance [21].

In contrast, if the bottleneck is deepened, such as an ECC block, the decrease in speed of the instruction fetch step degrades the overall performance [22,23]. In this case, it may be necessary to modify the overall architecture, which is not desirable. Therefore, it is necessary to eliminate bottlenecks and minimize performance degrading through the efficient design of the ECC block.

**Figure 3:**
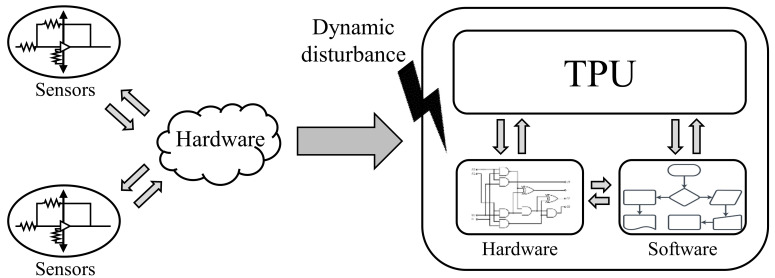
Dynamic disturbance of the smart sensor structure.

As shown in Figure 3, a smart sensor that collects and processes data from multiple sensors using built-in computing resources consists of several types of sensors and MCUs. Since the smart sensor has an MCU, there are small ups and downs to process the software. Since these smart sensors sense and process data in poor and noisy environments, data integrity needs to be guaranteed. In an environment where data integrity is not guaranteed, software-based connected sensors may have no effect or cause more serious errors. At the same time, the ECC to ensure the integrity of the smart sensor needs to be small and not affect the speed. Therefore, an efficient design of the ECC block is required.

This study is based on existing research, developed on the basis of the existing decoding process, and the encoding process was added, which led to faster execution results than the existing TPU. In addition, ECC is used in both memory writing, reading, and encoding processes so that more tests can be executed.

## 2. Background

The Hamming code is one of the most frequently used algorithms in embedded systems that require ECC. Since only additional bits are transmitted without repetitive transmission, a data rate above a certain level is guaranteed. In addition, since two or more errors rarely occur on average in the encoded data, the Hamming code that satisfies SECDED is the most efficient [24]. The Hamming code algorithm generates parity bits through the XOR operation of data bits, stores them together, and guarantees integrity through a decoding process before data are used. In addition, the number of parity bits ($p_{n}$) required increases as the size of the data ($d_{n})$ increases. The number of $p_{n}$s required to protect the required $d_{n}$ data using a Hamming code can be written as the inequality below.

(1) $$\begin{array}{c} 2^{p_{n}}\geq d_{n}+p_{n}+1 \end{array}$$

In this study, we used a 16-bit TPU, and according to the above inequality (1), $p_{n}$ should be 5-bit. In the encoding process, the parity bit $p_{n}$ is placed in the $2^{n}\left(n\geq 0\right)$ position. The input parity bit is generated by the XOR operation of data at a specific position. This specific data are created by reading and skipping as many as $2^{n}$ data from the position of $p_{n}$. From an example of the (7,4) Hamming code, we can see the operation of the algorithm. From Equation (1), a 3-bit parity bit is required for 4-bit data protection.

(2) $$\begin{array}{c} p_{0}=d_{0}⊕d_{1}⊕d_{3} \end{array}$$

(3) $$\begin{array}{c} p_{1}=d_{0}⊕d_{2}⊕d_{3} \end{array}$$

(4) $$\begin{array}{c} p_{2}=d_{1}⊕d_{2}⊕d_{3} \end{array}$$

Equations (2)–(4) are the process of generating 3 bits of parity bits to encode 4 binary data. The resulting data are extracted and used through the decoding process.

In the decoding process, the decoder determines whether the received data are corrupted, and if they are, they are corrected to obtain the original data. Otherwise, the original data are extracted without any modification. To check if the data have been corrupted, the decoder creates another parity bit ($p_{n}^{\prime }$) and syndrome ($S_{n}$). The syndromes are created as follows.

(5) $$\begin{array}{c} p_{0}^{\prime }=d_{0}⊕d_{1}⊕d_{3} \end{array}$$

(6) $$\begin{array}{c} p_{1}^{\prime }=d_{0}⊕d_{2}⊕d_{3} \end{array}$$

(7) $$\begin{array}{c} p_{2}^{\prime }=d_{1}⊕d_{2}⊕d_{3} \end{array}$$

(8) $$\begin{array}{c} S_{0}=p0⊕p^{\prime }0 \end{array}$$

(9) $$\begin{array}{c} S_{1}=p1⊕p^{\prime }1 \end{array}$$

(10) $$\begin{array}{c} S_{2}=p2⊕p^{\prime }2 \end{array}$$

The above Equations (5)–(10) represent the operations that take place in the decoder. The decoder creates a new parity bit from data and generates a syndrome through the XOR operation using the existing parity bit. Therefore, $S_{n}$ represents the location of the corrupted data, and the error is corrected by flipping the data at that location. Since the Hamming code we use is a SECDED code, an error of more than 2 bits cannot be distinguished from a single bit error. Flipping the wrong bit to the multi-bit error can lead to unexpected results. Therefore, additional bits can be used to detect multi-bits, but considering the load on the microprocessor, this study uses the SECDED Hamming code. Integrity is important for equipment that includes a small MCU, such as a TPU or smart sensor, but there are many considerations, such as design size, power consumption, and speed. ECC that can be mounted on TPUs or smart sensors in such various aspects as above is being studied extensively. As shown in Table 1, In the case of LDPC, it is used for 5th generation wireless communication, or is currently being used in many storage devices. In addition, it is also possible to correct the bit value even for various errors outside the SECDED code. However, the process of creating a check set shows a slightly more complex environment compared to the Hamming code. BCH is similar to LDPC, but shows high efficiency in a long code or very fast transmission environment [25]. In the case of CRC, it guarantees very high integrity, but additional design is required, such as a process of correcting errors in mathematical calculations or transmission failures, or requesting re-reception. On the other hand, the Hamming code has low integrity, but this is sufficient for MCUs such as TPUs and smart sensors. In addition, the Hamming code was adopted as ECC in this study because there are few factors that can reduce speed, and power consumption was also low due to the small design size.

There have been many studies to use ECC in Embedded, and many studies have focused on reducing the ECC load. [26,27,28] It is important to reduce the ECC load in the Embedded, where the operating environment is not good. Existing studies try to reduce the load by changing the memory structure [13] or remove the load through the modulator [29]. In this paper, we found the problems when ECC was added to Embedded and trIed to solve them through an on-chip encoder and decoder design. The RISC-based TPU is a processor with a limitation of hardware due to its design structure, and requires a lot of time to read data from the memory. Therefore, TPU follows the Harvard structure to use memory divided into data memory and instruction memory to solve the memory speed problem. However, the memory speed problem is not completely solved because it still has a von Neumann structure and Harvard structure at the same time. Memory bottlenecks in the von Neumann structure are often presented as an important factor [30]. Since the memory speed is slow compared to the operation speed of the ALU and register, it has several bottlenecks in reading and decoding instructions and sending control signals. To solve this problem, several studies have been conducted, such as using an external high-speed memory core or integrating a memory cell and a processor [31].

**Figure 4:**
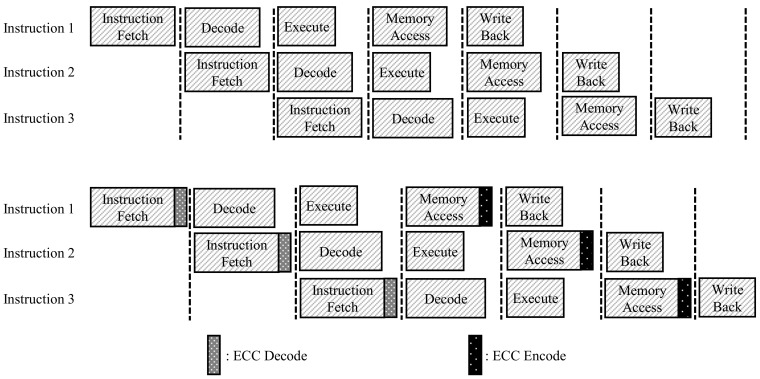
Deepened bottleneck.

In addition, since data must be extracted through the decoding process before the ALU step, ECC introduced to improve reliability reinforces the bottleneck [32], as shown in Figure 4, which significantly degrades the performance of the TPU. To use ECC in an embedded system, we have to consider performance, reliability, and memory usage. Recent research shows a lot of performance improvement thanks to parallel processing in embedded systems.

Previously, our research was conducted using only the ECC decoder [33]. In previous studies, a complete test was not possible due to the absence of an ECC encoder. Therefore, we developed the TPU by using an ECC encoder and decoder, and could reuse the processed data. In this study, we proposed a method for increasing memory usage by applying parallel processing to the decoding and encoding process of ECC, thereby improving performance.

## 3. Proposed Architecture

This section describes the parallel decoding module of the Hamming code, and the change of processing speed and memory usage of the proposed architecture. In the basic TPU without ECC, input and output processes of the data in the memory are often corrupted because of external shock or insufficient power supply. However, the added ECC decoder causes a severe bottleneck with a considerable amount of computation compared to a small embedded system. To solve this problem, we propose a method to debase the bottleneck by dividing the instruction into three parts and parallelizing the decoder.

A 16-bit machine with TPU’s instruction consists of a 4-bit OPCODE, 4-bit OPERAND1, and 8-bit OPERAND2. The instructions are divided into three parts as above, and are operated in parallel.

**Figure 5:**
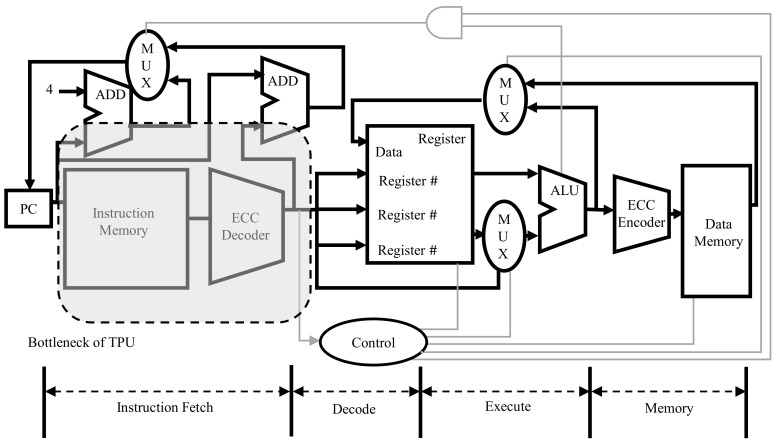
TPU with ECC decoder.

The TPU has five pipeline sections, and in the instruction fetch and write operand stages, the TPU should access instruction memory and data memory. Although several methods have been proposed for increasing the speed of these steps, the speed of accessing memory, storing, and fetching data are often a bottleneck in the TPU’s pipeline, as shown in Figure 5. In this situation, ECC decoding and encoding, which must operate simultaneously with memory, intensify the bottleneck. In this study, we propose a parallel processing method for ECC encoding and decoding of data and instructions.

**Figure 6:**
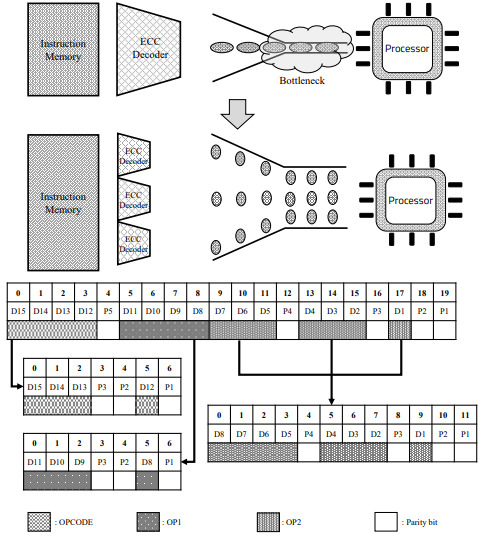
Encoding table bottleneck comparison of original and proposed models.

As shown in Figure 5, we added ECC encoders and decoders to the front and back of the memory to ensure the integrity of the storage process of the memory and data in the memory. Figure 6 shows the difference between the TPU structure with the basic ECC decoder added, and the TPU structure with the ECC parallel processing decoder added. As shown at the top of Figure 6, one large ECC decoder is located between the register and memory. It is in charge of continuously detecting and correcting errors by separating 21-bit data, including 5-bit ECC data and 16-bit real data, using one decoder, and creating a syndrome. However, the model we propose works by dividing the large decoder into three smaller decoders, as shown in Figure 6. Each decoder is responsible for opcodes and operands 1 and 2. It spreads out time-consuming tasks simultaneously. Detailed processing is as follows.

### 3.1. Proposed Encoding Process

The encoding process involves storing the data to be used in memory along with the ECC parity bits. Since it has not been done in previous studies, this paper discusses the process and method of encoding. Since the encoding process also accesses the memory, a bottleneck may occur when fetching instructions. Therefore, even when using ECC, it is necessary to prevent numerous tasks from being concentrated in one encoder in parallel. Unlike the previous 16-bit encoding as 21-bits using five parity bits, the proposed model is divided into three parts, as shown in Figure 6. There are the 4-bit opcode, 4-bit operand1, and 8-bit operand2 in the existing fixed instruction. Since each part is encoded separately, all encoding processes can be performed in parallel. However, in this process, more parity bits should be used than before. According to Equation (1), three, three, and four parity bits are required, respectively, unlike the existing five parity bits, and 10 parity bits are required to encode 16-bits into a Hamming code. This process is executed when the computed data are written back to memory, and parallelization and acceleration of the encoding process also help improve the performance of the TPU.

### 3.2. Proposed Decoding Process

The proposed ECC decoder structure aims to have the structure, as shown in Figure 6. Since the ECC decoding process is performed at the Instruction Fetch time, this intensifies the bottleneck of the TPU structure. Because of this, the bottleneck of the instruction fetch step is often intensified. The instruction contains an operand, the type of opcode and register to use. Since the command is already divided into three parts and encoded, the decoding process is simplified to some extent. The parity bit is extracted again for each of the three commands, and a syndrome is created to compare and find an error. For the existing model, one ECC decoder obtains 16-bit original data from 21-bit instructions, which takes a long time to decode the original data, and which degrades TPU performance. On the other hand, for the proposed model, we tried to reduce the bottleneck by dividing the instruction into three parts and processing each instruction in parallel. The location where each parity bit is generated and inputted is shown in Figure 6, as in the encoding process.

### 3.3. TPU Performance

As in Section 3.1 and Section 3.2, the reason why operation codes and operands can be executed in parallel is that each operation code and operands are operated independently. This allows the necessary signals to be selectively and quickly decoded first and then sent to the control module. For example, the opcode can be decoded first and used for the next pipeline operation. The opcode moves to the control module through one small parallel decoding module rather than the big one, as shown in Figure 5. This shortens the time to read commands that previously had bottlenecks and to send signals to the control module. This is an important key to reduce TPU bottlenecks. This can also help pipeline optimization by reducing the time spent in bottleneck. However, as described in Section 3.1 and Section 3.2, the amount of memory usage increases by a certain amount. This does not affect the instruction fetch time because the bandwidth of the memory can be adjusted.

In addition, the reason that the proposed model uses more memory is that it uses more parity bits, as shown in the downside of Figure 6, and the instruction is also split, so more parity bits must be used. This has the effect of increasing the reliability and the stability of TPU because many parity bits protect data, even though the instruction is longer.

### 3.4. Memory Usage and Size of Logic

In the proposed model, as shown in Figure 6, three parity bits are required for the opcode, and three and four parity bits are required for op1 and op2, respectively. Since the proposed structure is operated by dividing an instruction into several parts, the ECC data required per instruction is increased from 5-bit to 10-bit. In addition, three decode modules are required to verify ECC. Since this generates 10 syndromes and performs ECC decoding, the size of the circuit may also increase. Compared with the existing model, 5 parity is required, but in the proposed model, a total of 10 parity bits are required for one instruction. This requires 21-bits and 26-bits per instruction to send 16-bit data. As a result, the data rate decreases from 80% to 61%. The data rate means the ratio of the actual data excluding the ECC parity bit among the total data. The existing model with an 80% data rate has 16-bit actual data out of 21-bits, and the proposed model with a 61% data rate has 16-bit actual data out of 26-bits. If the bandwidth is not properly designed, it may increase the memory read and write time. However, the use of several ECC data improves the accuracy and reliability of ECC.

## 4. Experiment

**Figure 7:**
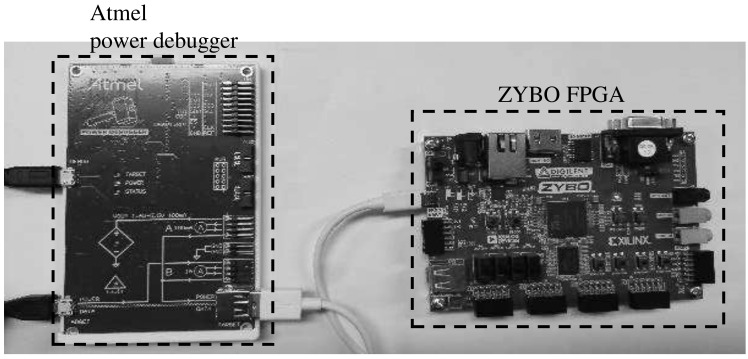
Experiment setup.

In this study’s experiment, we assumed the von Neumann bottleneck and used RISC-based self-made TPU, which was FPGA-tested using the “ZYBO” board. To derive the actual results, the decoding and encoding process was verified using “Design Compiler”, and we conducted the FPGA test using the actual “ZYBO” board, as shown in Figure 7. TPU is a RISC-V-based processor, and the TPU used in this paper uses a 16-bit instruction set. The TPU uses two separate memories, and the TPU has a pipeline stage of five stages and an execution speed of about 0.5 MIPS/MHz.

In this section, the TPU structure proposed in this paper was implemented, and experiments and comparative experiments were performed on the basic structure. TPU is emulated at the C++ level. We made it possible to measure the time consumed by each pipeline step, and we conducted several experiments, such as comparing the instruction execution speed of the TPU equipped with the decoder we propose. However, for more accurate experiments and measurements, the TPU of the proposed structure was implemented using Verilog, and “SIMVISION” was used for simulation. In addition, the synthesis of the generated decoder was performed using “Design Compiler.” Finally, an experiment was conducted to measure the performance speed and power consumption of the TPU using the FPGA.

To experiment by executing multiple instructions on the TPU, the product of the 3 × 3 matrix was transformed into a binary code and then repeated several times. On the same test bench, experiments were conducted to compare the speed and accuracy of each TPU equipped with each ECC decoding module.

**Figure 8:**
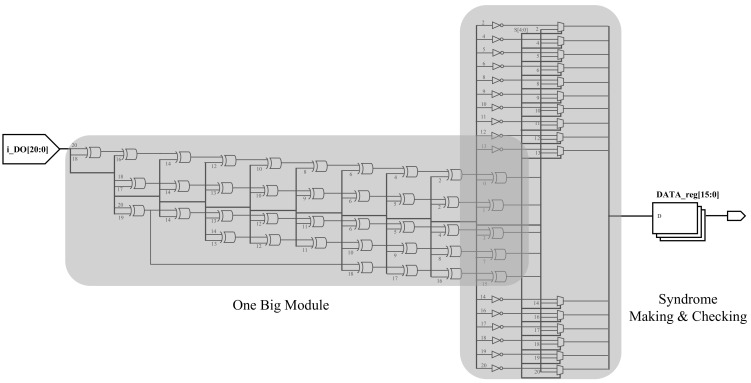
RTL synthesis of TPU with the ECC model.

**Figure 9:**
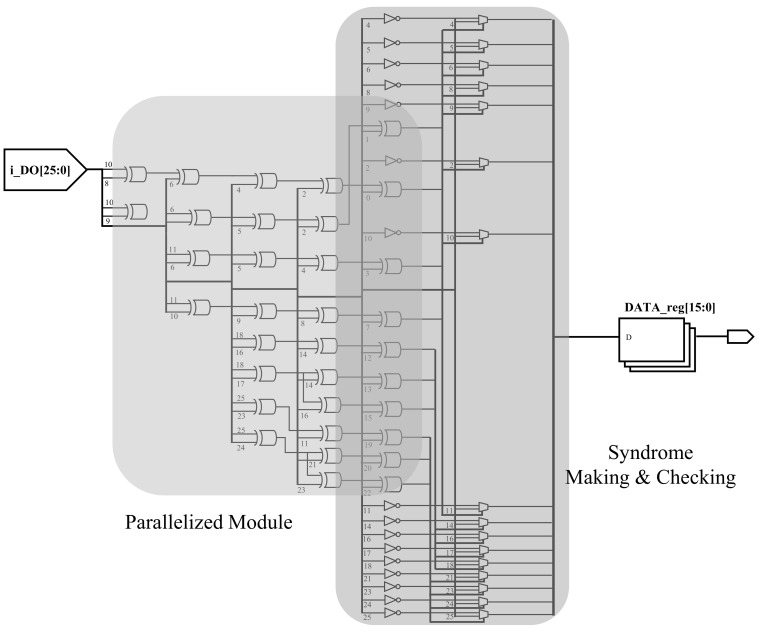
RTL synthesis of TPU with the proposed ECC model.

Figure 8 shows the existing model’s RTL synthesis result, and Figure 9 shows the proposed model’s synthesis result. Comparing Figure 8 with Figure 9, it can be seen that parity bits are generated through one big module. On the other hand, in Figure 9, parity bits are generated through a short path. As this task is repeatedly executed, there are differences in speed, area, and power consumption between Figure 8 and Figure 9. The synthesis results confirm that the decoder module was parallelized and the total area was increased.

### 4.1. Performance

**Figure 10:**
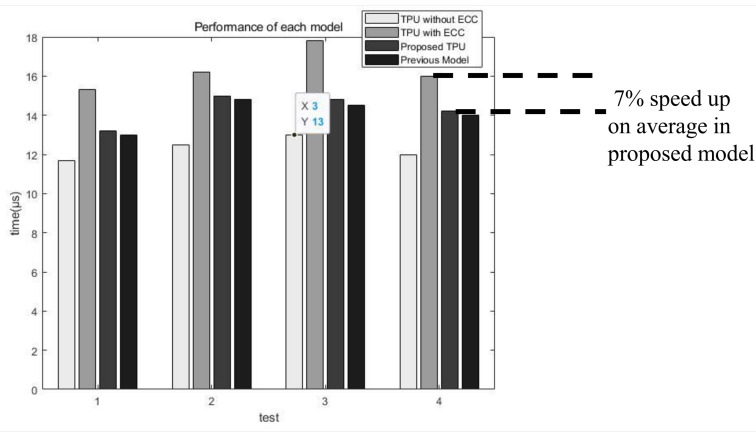
Execution speed of each model.

For the experimental data, a test bench was used in an environment in which the iterative statement for matrix multiplication and addition multiplication were mixed. We have seen some performance changes, including an encoding process that was not done in our previous paper. In the previous study, because the encoded data was used, there was a difference in speed due to the time taken during the encoding process. When ECC is applied, the original Hamming ECC model shows a speed reduction of approximately 30% compared to the system without ECC. This is because there is no need to apply ECC in processing, decoding, or encoding data, where there is no ECC model. However, the proposed ECC parallel processing decoder model increases performance by 7% compared to the existing ECC decoder, shown in Figure 10. The addition of the proposed decoder minimizes the performance degradation of the TPU. In the case of TPU, since it operates by pipelining, the speed improvement of decoder and encoder does not lead to overall TPU performance improvement. Therefore, a 30% speedup of the proposed decoder leads to 7% of the TPU performance.

The proposed parallel ECC processing process results in approximately 7% faster speed than the existing ECC processing process. In addition, the result of testing the TPU by introducing this decoder is as follows. It took 12.5 μs to perform all the test benches in the existing TPU, and 16.2 μs was consumed in a non-parallelized structure. Finally, the proposed structure with a parallelized decoding process consumes 15 μs. Because of several test benches, a speed improvement of approximately 7% is achieved compared to the conventional ECC TPU model numerically. This is the result of removing the bottleneck in the decoding process and allowing a faster clock to be used. Compared to the TPU equipped with the existing decoder, the TPU equipped with the proposed decoder can use a clock that is about 10% faster by removing the bottleneck.

### 4.2. Reliability

**Figure 11:**
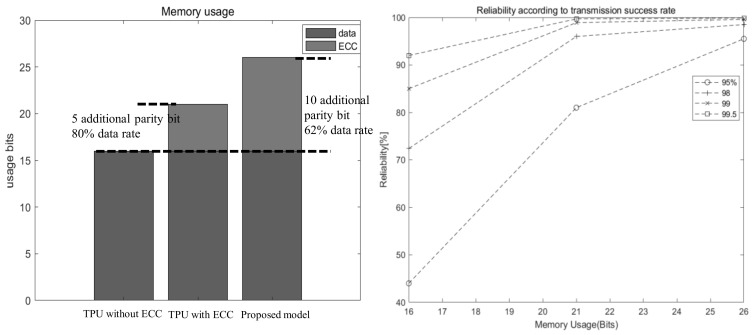
Memory usage and reliability of each model.

ECC improves reliability when the data transfer success rate is not high in the TPU. In short, the proposed model provides better reliability compared to existing models in harsher environments. The situation in Table 2 assumes that the transmission success rate is extremely low. If the probability of actually transmitting 1 bit is 95%, the proposed model achieves a 16-bit transmission success rate of 95.5%, 17% higher than that of the existing ECC model. This decreases as the transmission success rate increases, but it shows that it guarantees sufficiently high reliability compared to the existing model. Table 2 shows the improvement of TPU stability according to each transmission success rate. The proposed model achieves better reliability by introducing an additional parity bit in the existing model. The values in Table 2 and Figure 11 are obtained through the equation below.

(11) $$Transmission\;success\;rate/instruction\left(\%\right)={success\;rate/1bit}^{N}+{success\;rate/1bit}^{N-1}*N$$

It shows that the proposed model can be efficiently used in an environment where a transmission success rate is lower than 99%.

### 4.3. Memory Usage Comparison

In the proposed model, since the instruction is divided into three parts to create and store the ECC code, a larger amount of parity bits is required compared to the existing model. Compared with the conventional 5-bits parity bit in the 16-bit TPU, the proposed model requires 10-bits, and thus the data required for 16-bit data transmission is from 21-bit to 26-bit, as shown in Figure 11. As it increases, the data rate drops from 80% to 61%. Notably, the memory of the proposed model increases by 23% compared to the conventional memory numerically. This is a part that can place a load on the microprocessor, and this must be solved by additionally using an ECC dedicated memory.

### 4.4. Cell Area

Since the decoder and encoder use the proposed model process data in parallel, the overall design area is increased. As a result of the design, the ECC decoder shows an area increase of approximately 6% compared to the original design. In addition, the encoder shows an area increase of approximately 8%. This appears to be a phenomenon that the syndrome increases for parallel processing, because of an increase of the parity bit. The encoder and decoder sizes increase, but in terms of the overall TPU, this is a very small size increase.

### 4.5. Power Consumption

**Figure 12:**
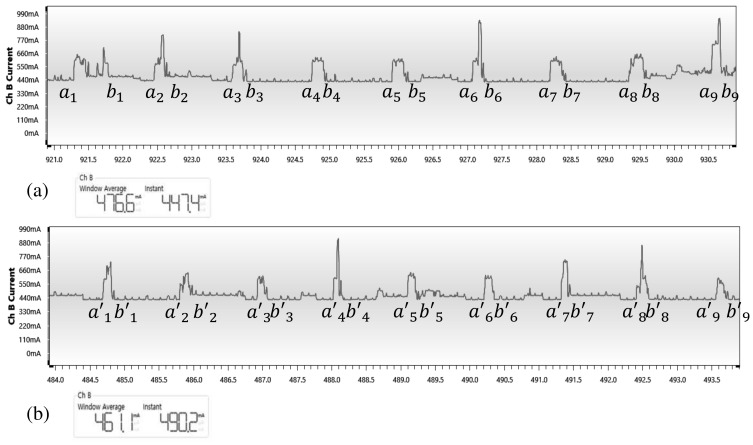
(**a**) Power consumption of original model. (**b**) Power consumption of proposed model.

Power consumption is one of the most important considerations in the design of an embedded system. In this study, we experimented with “Atmel Power Debugger” and “Zybo z7” boards. To compare the power consumption of the original model and proposed model, the decoding process was repeated 100 times per each clock. As shown in Figure 12, the original model takes 476.6 mA of the current on average, and the proposed model takes 461.1 mA of the current on average. Peak current consumption in the decoding process is almost the same, but the proposed model reduces execution time through improved performance of 7% compared to the original ECC decoder.

According to Figure 12, the used energy of decoding processes are as follows [34]. ’A’and ’b’ means the operation start and end time of the existing decoder model, and a’ and b’ mean the operation start and end time of the proposed decoder model, respectively.

(12) $$E_{original}=\int_{a_{1}}^{b_{9}}P_{i}\left(t\right)-P_{standby}\;dt$$

(13) $$E_{parallel}=\int_{a_{1}^{\prime }}^{b_{9}^{\prime }}P_{i}\left(t\right)-P_{standby}\;dt$$

To calculate each of the Equations (12) and (13), it can be expressed as below.

(14) $$\int_{a_{1}}^{b_{9}}P_{i}\left(t\right)-P_{standby}\;dt=\sum_{n=1}^{9}\left(b_{n}-a_{n}\right)*P_{average}$$

(15) $$\int_{a_{1}^{\prime }}^{b_{9}^{\prime }}P_{i}\left(t\right)-P_{standby}\;dt=\sum_{n=1}^{9}\left(b_{n}^{\prime }-a_{n}^{\prime }\right)*P_{average}$$

The energy consumed in the decoding process is achieved by the above Equations (14) and (15), which is determined by the difference between ($b_{n}^{\prime }-a_{n}^{\prime }$) and ($b_{n}-a_{n}$).

(16) $$\left(b_{n}^{\prime }-a_{n}^{\prime }\right)<\left(b_{n}-a_{n}\right)$$

Due to the increased performance of the proposed model, as shown in inequality (16), the ($b_{n}^{\prime }-a_{n}^{\prime }$) time is shorter than ($b_{n}-a_{n}$). This shows that the increase in speed due to parallel execution of ECC leads to a decrease in the energy consumption of the processor. Looking at the results measured using the actual FPGA, since the FPGA operates with 5 V power, the conventional decoder uses about $0.6\times 10^{-3}\left[J\right]$ per operation, and the proposed model consumes $0.522\times 10^{-3}\left[J\right]$ of energy. This is a result of shorter running times and lower power consumption.

## 5. Discussion

The proposed model and the existing models have been examined in terms of speed, memory usage, reliability, and cell area. The proposed model achieved sufficient speed and reliability improvement. The proposed model achieved 7% speed improvement through parallel processing of ECC compared to the model using the existing ECC. In addition, the use of several parity bits and syndromes also increased reliability. This is related to improved performance and robust execution, which are important in embedded systems.

The proposed model has shown applicability in several embedded systems that may have additional ECC memory. Although additional memory is used, it is easy to use because it does not change the original data, and it easily guarantees data integrity. This technique can be used not only for TPUs, but also for memory transmission, and in this case, it can be used by increasing the memory bandwidth.

In terms of circuit size and power consumption, it is inevitable to increase the size of the circuit because three decoders and encoders are used. In the decoder and encoder parts, there is a certain amount of increase in size, but this seems to result in a slight change in the overall area. Power consumption needs to be considered when using an embedded system. The change in power consumption is related to the change in speed. Since the proposed decoding model has improved speed compared to the existing decoding model, the overall execution time and energy consumption are reduced. Though overall memory usage increases, it may be necessary to increase the instruction memory.

In addition, if the transmission error rate is high when longer instructions with ECC are loaded, additional errors may occur accordingly. To overcome these limitations, an additional ECC memory storing ECC parity bits is required. It needs to solve the memory usage problem by using more stable ECC memory and to guarantee the integrity between data transmission and reception.

## 6. Conclusions

In this study, we proposed a decoder and encoder block parallelization structure to minimize the bottleneck that occurs when applying the Hamming code ECC in a TPU having both von Neumann and Harvard structures and to improve the reliability of the TPU. When the proposed structure was applied to the TPU, the use of additional memory improved speed and reliability. Experimental results confirmed that a negligible increase in size and power is achieved by using the proposed model. In contrast to the model without ECC, an additional ECC of 10 bits per instruction is required for the model, and an additional parity bit of 5 bits per instruction is required compared to the existing ECC model. This needs 23% of additional memory compared to the previous model; however, the speed increase and reliability improvement by 7% were verified. The proposed model shows that if sufficient memory is provided, fast and stable instruction execution is possible simultaneously by using additional memory, so it can be applied to an embedded system in which there is insufficient power supply and requiring high speed and stabilization. In this paper, ECC, which was often processed through existing software, was implemented in TPU through the hardware design on the OnChip. In addition, the problem of a deep bottleneck resulting from this result can be quickly dealt with through separate encoders and decoders. Additionally, by increasing the memory used, the reliability was improved. It leads to the integrity and reliability of critical data in the OnChip design, as well as an increase in speed with the use of additional memory. This is a method that can be easily applied to other chips, and the validity of the chip can be obtained by using additional memory. It is expected that this will be a valuable contribution by improving the speed and reliability of embedded chips, which are the most important in the fourth industrial revolution. Performance and reliability in AI chips or autonomous driving chips in the fourth industry are the most important points. It is expected that the ECC encoder and decoder of the model proposed in this paper can contribute a lot to the above chips. In order for the concept of this paper to be applied to an actual chip, it is necessary to understand the bottleneck of the actual chip. Because the chip often has a lot of bottlenecks due to memory speed, in this case, this study can be very helpful. In addition, the parallel decoding method presented in this study is expected to be used in many processors with a speed limit in the instruction patch stage with a von Neumann structure.

## Figures and Tables

**Table 1:** Comparison of several ECC for TPU or smart sensors.

Type of ECC	Hamming Code	LDPC	BCH	CRC
Integrity	low	middle	middle	high
Speed	fast	middle	middle	low
Design Size	small	middle	middle	big
Power Consumption	low	middle	middle	big

**Table 2:** Reliability according to the transmission success rate.

Transmission Success Rate of 1 Bit	95%	98%	99%	99.5%
TPU without ECC	44%	72.4%	85%	92%
TPU with Original ECC	81%	96%	98.9%	99.7%
TPU with Proposed Model	95.5%	98.5%	99.6%	99.9%
Rate of Rise Compared to Original ECC	17%	2%	0.7%	0.2%

## Abbreviations

The following abbreviations are used in this manuscript:

RISC	Reduced Instruction Set Computer
ECC	Error Correcting Code
TPU	Tiny Processing Unit
SECDED	Sing Error Correction Double Error Detection
ALU	Arithmetic Logic Unit
SOTA	State Of The Affairs
